# Retrospective French nationwide survey of childhood aggressive vascular anomalies of bone, 1988-2009

**DOI:** 10.1186/1750-1172-5-3

**Published:** 2010-02-03

**Authors:** Sébastien Héritier, Martine Le Merrer, Francis Jaubert, Michèle Bigorre, Marion Gillibert-Yvert, Benoit de Courtivron, Makram Ziade, Yves Bertrand, Christian Carrie, Pascal Chastagner, Cécile Bost-Bru, Jean-Claude Léonard, Marie Ouache, Liliane Boccon-Gibod, Pierre Mary, Jacques de Blic, Isabelle Pin, Daniel Wendling, Yann Revillon, Véronique Houdoin, Véronique Forin, Hubert Ducou Lepointe, Jane Languepin, Jeanne Wagnon, Ralph Epaud, Brigitte Fauroux, Jean Donadieu

**Affiliations:** 1 Service d'hématologie oncologie pédiatrique, Centre de référence des histiocytoses, AP-HP Hôpital Armand Trousseau, Paris, France; 2 Service de génétique, Centre de référence des maladies osseuses, AP-HP Hôpital Necker, Paris, France; 3 Service d'anatomie et cytologie pathologique, AP-HP Hôpital Necker, Paris, France; 4 Service de chirurgie plastique pédiatrique, Hôpital Lapeyronie, Montpellier, France; 5 Service d'orthopédie, Hôpital Clocheville, CHU de Tours, France; 6 Institut d'hématologie et d'oncologie Pédiatrique, Lyon, France; 7 Département de radiothérapie, Centre Léon Bérard, Lyon, France; 8 Service de Médecine Infantile, Hôpitaux de Brabois, Vandoeuvre les Nancy, France; 9 Pédiatrie, Pôle Couple enfant, CHU de Grenoble, France; 10 Institut Calot, Berck sur Mer, France; 11 Service d'hématologie, AP-HP Hôpital Robert Debré, Paris, France; 12 Service d'anatomie et cytologie pathologique, AP-HP Hôpital Armand Trousseau, Paris, France; 13 Service d'orthopédie, AP-HP Hôpital Armand Trousseau, Paris, France; 14 Service de pneumologie, AP-HP Hôpital Necker, Paris, France; 15 Service de rhumatologie, CHU de Besançon, France; 16 Service de chirurgie viscérale, AP-HP Hôpital Necker, Paris, France; 17 Service de rééducation, Centre de référence des maladies osseuses, AP-HP Hôpital Armand Trousseau, Paris, France; 18 Service de radiologie, AP-HP Hôpital Armand Trousseau, Paris, France; 19 Service de pédiatrie générale, CHU de Limoges, France; 20 Service de pédiatrie générale, CH de Morlaix, France; 21 Service de pneumologie, Centre de référence des maladies pulmonaires rares, AP-HP Hôpital Armand Trousseau, Paris, France

## Abstract

**Objective:**

To document the epidemiological, clinical, histological and radiological characteristics of aggressive vascular abnormalities of bone in children.

**Study design:**

Correspondents of the French Society of Childhood Malignancies were asked to notify all cases of aggressive vascular abnormalities of bone diagnosed between January 1988 and September 2009.

**Results:**

21 cases were identified; 62% of the patients were boys. No familial cases were observed, and the disease appeared to be sporadic. Mean age at diagnosis was 8.0 years [0.8-16.9 years]. Median follow-up was 3 years [0.3-17 years]. The main presenting signs were bone fracture (n = 4) and respiratory distress (n = 7), but more indolent onset was observed in 8 cases. Lung involvement, with lymphangiectasies and pleural effusion, was the most frequent form of extraosseous involvement (10/21). Bisphosphonates, alpha interferon and radiotherapy were used as potentially curative treatments. High-dose radiotherapy appeared to be effective on pleural effusion but caused major late sequelae, whereas antiangiogenic drugs like alpha interferon and zoledrenate have had a limited impact on the course of pulmonary complications. The impact of bisphosphonates and alpha interferon on bone lesions was also difficult to assess, owing to insufficient follow-up in most cases, but it was occasionally positive. Six deaths were observed and the overall 10-year mortality rate was about 30%. The prognosis depended mainly on pulmonary and spinal complications.

**Conclusion:**

Aggressive vascular abnormalities of bone are extremely rare in childhood but are lifethreatening. The impact of anti-angiogenic drugs on pulmonary complications seems to be limited, but they may improve bone lesions.

## Introduction

Aggressive vascular abnormalities of bone consist of intra-osseous vascular abnormalities leading to osteolysis and, sometimes, extension to adjacent tissues [1]. These diseases are very rare, and affect children and young adults [2]. They may be disseminated or focal, but both forms can be very aggressive and life-threatening. These bone vascular abnormalities are distinct from malignant bone tumors with vascular proliferation, such as angiosarcoma and hemangioendothelioma [3]. In the literature, such entities are variously named "disseminated cystic bone angiomatosis" (mostly multifocal) [4] and "aggressive massive osteolysis" [5] (also called Gorham-Stout disease if unifocal). Some authors use the name Gorham-Stout disease for multifocal cystic bone angiomatosis [6-12], and nearly ten other names have also been used. A recent review of 43 cases of vascular lesions of bone recommended the use of the ISSVA classification [3].

The natural history of such diseases is mainly known through individual case reports, and there are only two series with more than 10 patients (15 and 11 cases)[13,14]. The lack of epidemiological data, together with the conflicting nature of available information, especially on the natural history and patient management, led us to conduct a retrospective study of cases diagnosed in France during the past 21 years. Here we report the epidemiological, clinical and biological characteristics, treatment modalities and outcome.

## Patients and Methods

All children (age <18 years at diagnosis) with a diagnosis of aggressive vascular abnormalities of bone (including "bone angiomatosis" and "Gorham-Stout disease") between 1 January 1988 and 1 September 2009 and managed in a French hospital were eligible for the study. We contacted all pediatricians belonging to the Society of Pediatric Hematology-Immunology and the French Pediatric Cancer Society, including orthopedic surgeons in French teaching hospitals. The diagnosis of aggressive vascular abnormalities of bone was based on histological examination of a bone lesion. A presumptive diagnosis could be made in the following circumstances: Situation A: bone osteolysis and chylous pleural effusion, with or without pulmonary lymphatic infiltration; and situation B: isolated bone osteolysis, provided Langerhans histiocytosis and infectious bone abscess were ruled out. In this latter case the diagnosis was based on three criteria: 1) the site of involvement (vertebra, ribs, pelvis and scapular belts); 2) few if any bone symptoms, in the absence of complications (fracture, including vertebral collapse); and 3) the following characteristic aspect on roentgenography or magnetic resonance imaging (MRI): well-defined piecemeal lesions; centromedullary and cortical involvement with no periosteal reaction; pseudocystic aspect and ring-shaped peripheral contrast uptake with secondary filling. In the absence of histological documentation, patients with osteolysis involving solely the feet and hands, and patients with associated renal disease [15], were excluded, as were patients with isolated pulmonary chylothorax and lymphangiectasis.

Multifocal osseous forms are defined as those in which osteolysis affects several bone segments distant from one another, with healthy bone in-between. Localized osseous forms are defined as those in which osteolysis affects only one bone with contiguous lesions, or several anatomically contiguous bones.

The date of diagnosis was the date of biopsy when relevant. For presumptive diagnoses, the date of diagnosis was the date of X-ray examination showing typical lesions. The date of first symptoms was the date when the first manifestations potentially related to the disease were reported by the patient or the family. Clinical remission is defined as the absence of any complication for at least two years. The following criteria were used to evaluate therapeutic efficacy: i) symptomatic lesions (dyspnea due to pulmonary involvement, clinically assessable cutaneous, subcutaneous or soft-tissue involvement): efficacy was judged at 3 months, based on changes in respiratory status or stabilization of lesion volume; ii) in the absence of symptomatic lesions (solely radiological signs of bone involvement or asymptomatic involvement of a deep organ [spleen]), efficacy was judged at 2 years, based on the occurrence of complications. Complications were defined as follows: bone fractures; vertebral collapse; consequences of tissue destruction due to proliferation of pathological tissue and necessitating hospitalization; and all other serious complications. Serious complications included disseminated intravascular coagulation; respiratory failure due to pleural effusion, or lung lymphangiectasis and vertebral collapse with neurological complications.

The following data were systematically collected from the patients' files: clinical information, i.e. personal and family history, symptoms; histology; biology; and imaging studies (including radiography of the lesions, MRI, scintigraphy, and bone densitometry). Treatments and complications were recorded from diagnosis to the last visit. No further examinations were requested for this solely observational study. Cases #10, #12 and #13 have been partially reported elsewhere [16-18]. The Kaplan-Meier method was used to estimate survival rates. The endpoint for the survival analysis was death. The period taken into account was the interval between the date of diagnosis and the event (or the last examination when no event occurred). The cut-off date was 15 September 2009.

## Results

### Characteristics of the patients

There were 8 girls and 13 boys Table S1, Additional file 1. Two children originated from North Africa (including a French resident), one was from Italy, and the other 18 children were of French origin. No parental consanguinity was found, and there was no known family history of aggressive vascular abnormalities of bone, or other vascular abnormalities. Patient #1 had had a hemangioma during infancy that regressed spontaneously. Two children were born prematurely (30 and 34 weeks). Patient #18 had a dominant form of familial exostosis, as did his mother. This patient was mentally retarded and had had three severe bacterial infections (pneumococcal meningitis, osteomyelitis due to betahemolytic streptococci, and *Staphylococcus aureus *pneumonia). Patient #12 was also mentally retarded and patient #7 had partial epilepsy.

Mean age at diagnosis was 8.0 years [0.8 to 16.9 years]. No clear triggering factors were identified. Two patients had suffered trauma close to the affected areas, more than two years previously. Four other patients had had infections in the region of the affected areas: patient #16 contracted mumps less than two months before onset, which was revealed by laterocervical tumefaction; patient #10 had had pleurisy and pneumococcal purulent pericarditis at age 4 years (9 years before the diagnosis of thoracic angiomatosis); patient #18 had had bilateral lymphatic pleural effusion after an episode of pneumonia associated with *Staphylococcus aureus *bacteremia; patient #3 had recurrent purulent meningitis (due to *Haemophilus influenza*e), revealing a meningeal defect at the third episode, leading to the diagnosis of aggressive vascular abnormalities of bone.

### Diagnosis and clinical manifestations

Pathological examination confirmed the diagnosis in 16 cases, on a sample of a bone lesion in 15 cases and of involved skin and spleen tissue in a patient with bone lesions. In three cases (#1, #3 and #7), a second biopsy was required for diagnosis. In case #9 an aggressive vascular abnormality of bone was diagnosed after re-examining the initial specimen, 6 years after misdiagnosis of Langerhans histiocytosis. In case #4 and #20 the only bone biopsy was non contributory. In cases #8 and #11, two biopsy procedures were complicated by heavy bleeding (including a case of pulmonary hemorrhage) and the diagnosis remained presumptive. These two cases were diagnosed on the basis of a combination of typical bone lesions and pleural effusion or lymphangiectasis on pulmonary CT, corresponding to presumptive diagnosis (situation A). In cases #2, #4 and #20 the diagnosis remained presumptive (situation B).

The median interval between the first clinical manifestations and the diagnosis of aggressive vascular abnormalities of bone, assessable in 18 patients, was 0.47 years (range 0.01-10.3 years). In nine cases, at least 6 months elapsed between the discovery of osteolysis and the diagnosis of aggressive vascular abnormalities of bone. Three patients initially received specific chemotherapy (vinblastine and steroids) for suspected Langerhans histiocytosis, to no effect. The first sign of the disease was an acute complication in 13 of the 21 patients, consisting of pathological fractures (n = 4), cyphosis due to vertebral collapse (n = 1), dyspnea (n = 7) due to abundant pleural effusion (n = 5) or to respiratory failure with pulmonary lymphangiectasis (n = 2), or meningitis due to an osteomeningeal breach (n = 1) [Table S1, Additional file 1]. One case was revealed by chronic lumbosacral pain and one by a persistent limp, while one case was diagnosed during investigation of chronic lumbago associated with growth retardation. Three patients presented with subcutaneous tumefaction and one case was diagnosed at age 7 years, during follow-up of a cervical lymphangioma, diagnosed at birth, which relapsed once at age 2 years and was then complicated by spleen involvement; later radiographs showed vertebral, scapular and iliac lesions. Finally, in one case the disease was diagnosed on radiographs prescribed for facial trauma following a vagal malaise.

### Extension

The two most frequently affected bone sites were the spine (16 cases, especially the dorsal spine), and the ribs (12 cases). The other sites, in order of decreasing frequency, were the pelvis, cranium, femur and humerus.

Sixteen patients had multifocal osseous forms at diagnosis. Six patients had more that 9 affected bone segments, including a child with 13 sites (case #6) [Table S1, Additional file 1]. Five patients had localized forms: two had lesions localized to the pelvis and lower limbs (cases #2 and #5) while the other three had lesions localized to the thoracic cage (ribs, dorsal spine and sternum; cases #10, 12 and #13).

**Figure 1:**
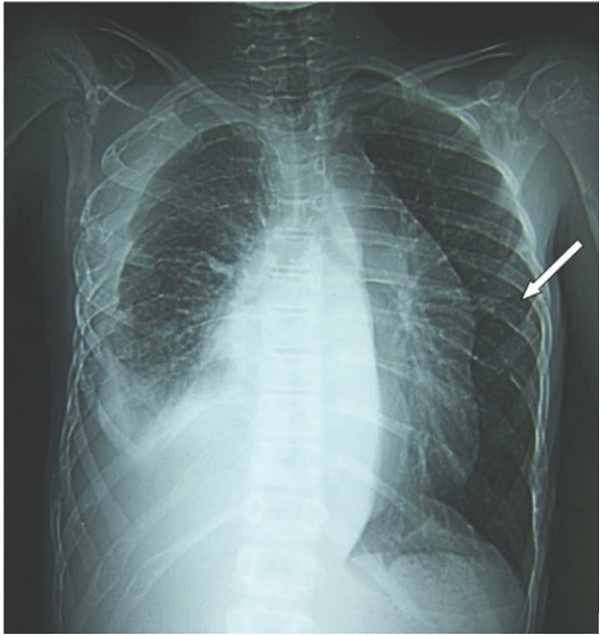
**Pulmonary involvement: pleural effusion associated with pulmonary lymphangiectasies (case #15) (White arrow: puffy aspect of costal cortical bone)**.

**Figure 2:**
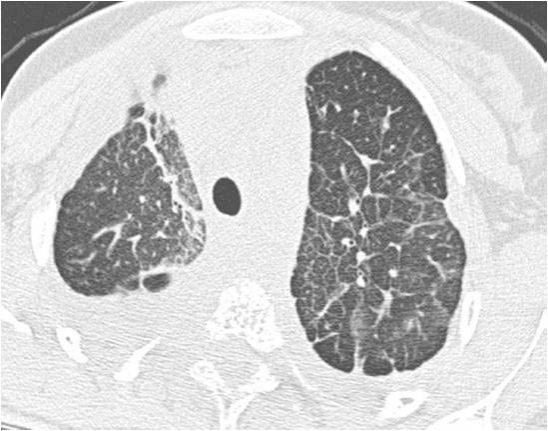
**Lung CT scan (case #8) showing lymphangiectasies**.

Extraosseous involvement was observed in 18 cases [Table S1, Additional file 1], usually at diagnosis. Pulmonary involvement occurred in 10 children: 9 children had pleural effusions (figure 1) composed of bloody fluid (n = 1), chylous fluid (n = 5) or initially hemorrhagic then chylous fluid (n = 1); in two cases the nature of the effusion was not determined. In five patients (#8, #10, #15, #18, #19) the pleural effusion was associated with pulmonary lymphangiectasies characterized radiologically by a diffuse interstitial syndrome (figure 2). Finally, one patient (#9) had a radiological interstitial syndrome related to pulmonary lymphangiectasies, but remained asymptomatic. Spleen involvement was diagnosed in 8 children and was always asymptomatic. The diagnosis was made on routine abdominal sonography showing cystic lesions. Cutaneous or subcutaneous involvement was reported in 7 cases. Patient #14 was found to have urinary bladder involvement at autopsy. Finally, many patients had soft tissue involvement close to their bone lesions, and patients #10 and #15 had extension to the mediastinum or peritoneum.

Among the five patients with localized forms, the three who had lesions localized to the thoracic cage developed lung involvement.

### Imaging and laboratory findings

**Figure 3:**
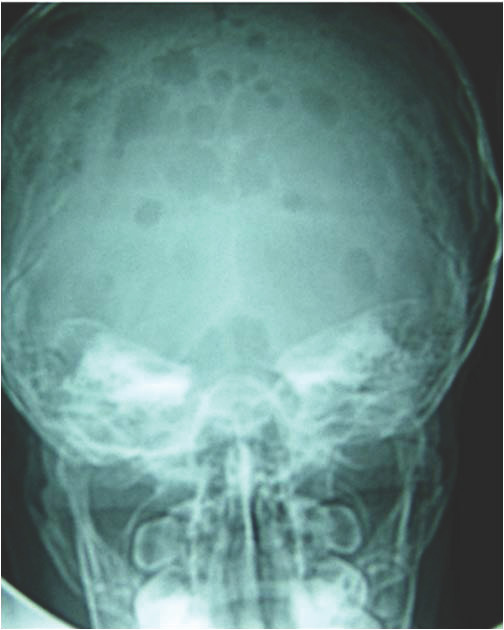
**Skull radiography with rounded well-defined radiolucent bone lesions (case #15)**.

**Figure 4:**
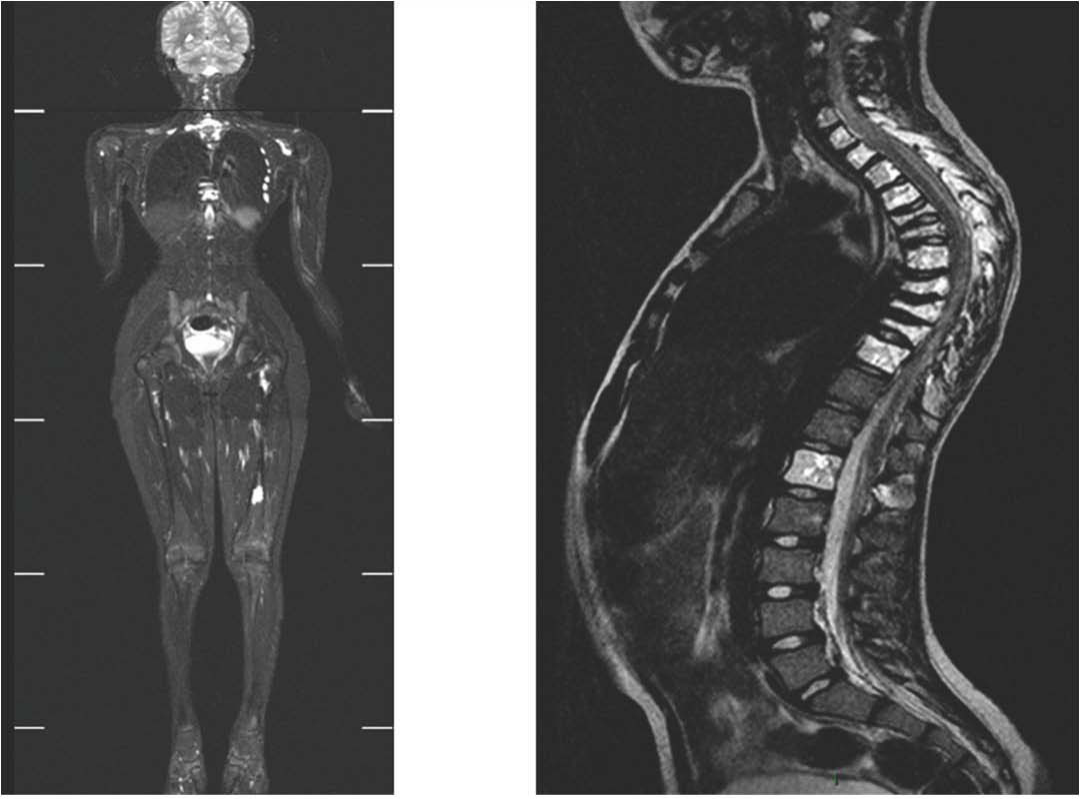
**Whole body coronal and sagittal MRI (case #20)**. T2-weighted MRI of spinal (C2; C3; C6→T10; L1), costal, left scapular and left femoral lesions.

Patients with multifocal osseous forms had rounded well-defined radiolucent bone lesions on imaging studies (figure 3). Rib involvement gave a puffy aspect to the cortical bone (figure 1), which was very similar in the five relevant cases (#1, #7, #9, #13, #15). MRI findings consisted of well-defined bone lesions that were T1 hypointense and T2 hyperintense (figure 4) and took up contrast medium, giving an aspect of cystic bone lesions. In 10 of the 11 cases in which the bone matrix was studied, the aspect was one of a general decrease in skeletal bone density, confirmed by bone densitometry (≥ 3 SD). Technetium scintigraphy (n = 7) failed to detect the bone lesions and was far less sensitive than MRI, bone scan or radiographs. Regarding biological findings, only patient #18 had an inflammatory syndrome. The water-electrolyte balance and hepatic biochemistry were normal in all the patients. Five patients (cases #8, #10 # 11 #18 and #19) had biological signs of disseminated intravascular coagulation (DIVC). These five patients had concurrent progressive pulmonary involvement (symptomatic pulmonary lymphangiectasis in two cases, and pleural effusion in three cases). Patient #18 had lymphopenia (between 400 and 600/mm^3^) without hypogammaglobulinemia, No immune deficiency was observed in the three patients who initially presented with a bacterial infection.

### Pathological findings

**Figure 5:**
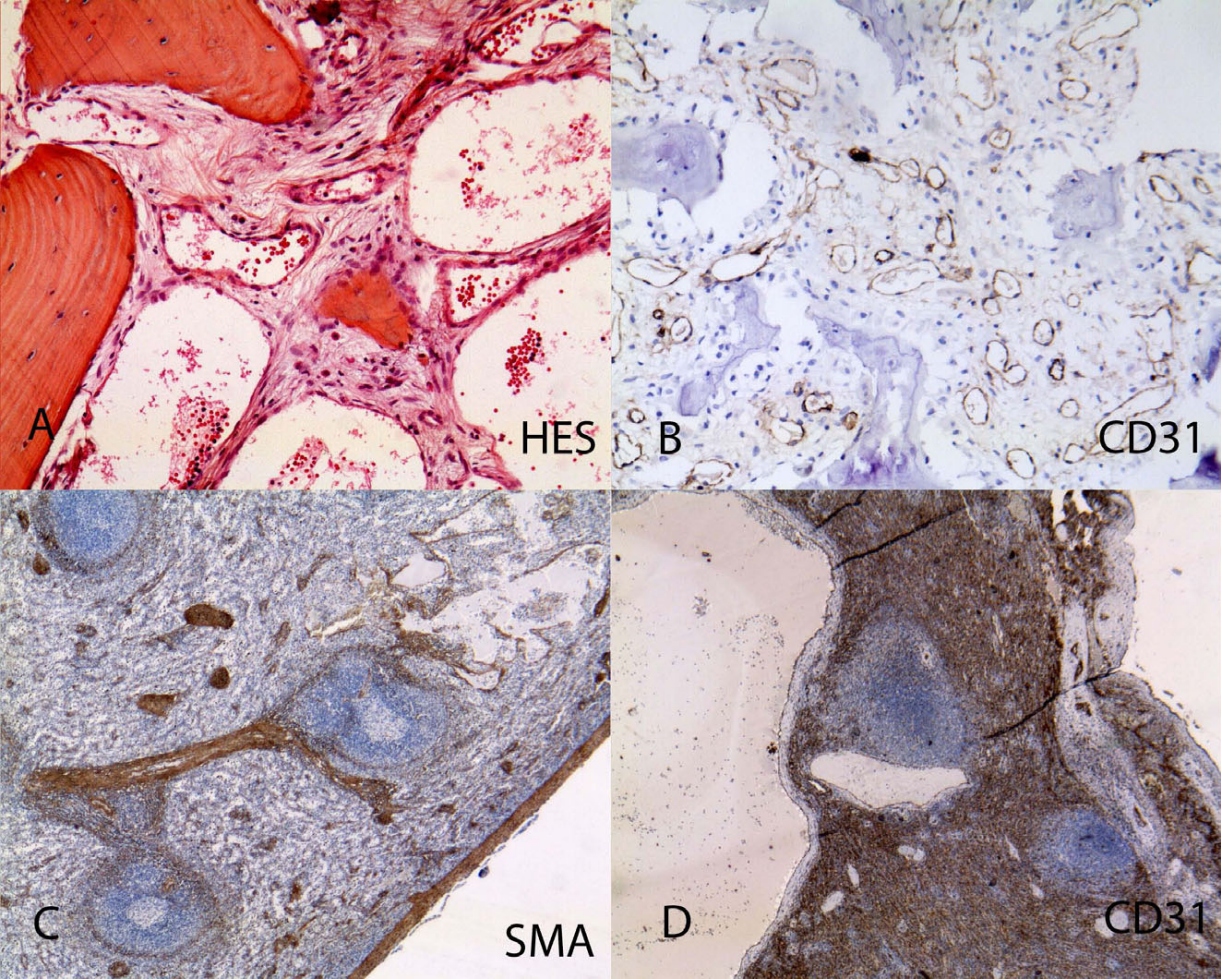
**Pathological findings: Hematopoietic locations**. A: Capillarized sinusoids of a bone biopsy with an inflammatory surrounding (HES ×400). B: CD31 positivity of the endothelial lining of capillarized sinusoids (×200). C: Spleen dilated sinusoids (smooth muscle actin ×100). D: Spleen larger cysts lined by a continuous layer of CD31 endothelial cells (CD31 × 100).

**Figure 6:**
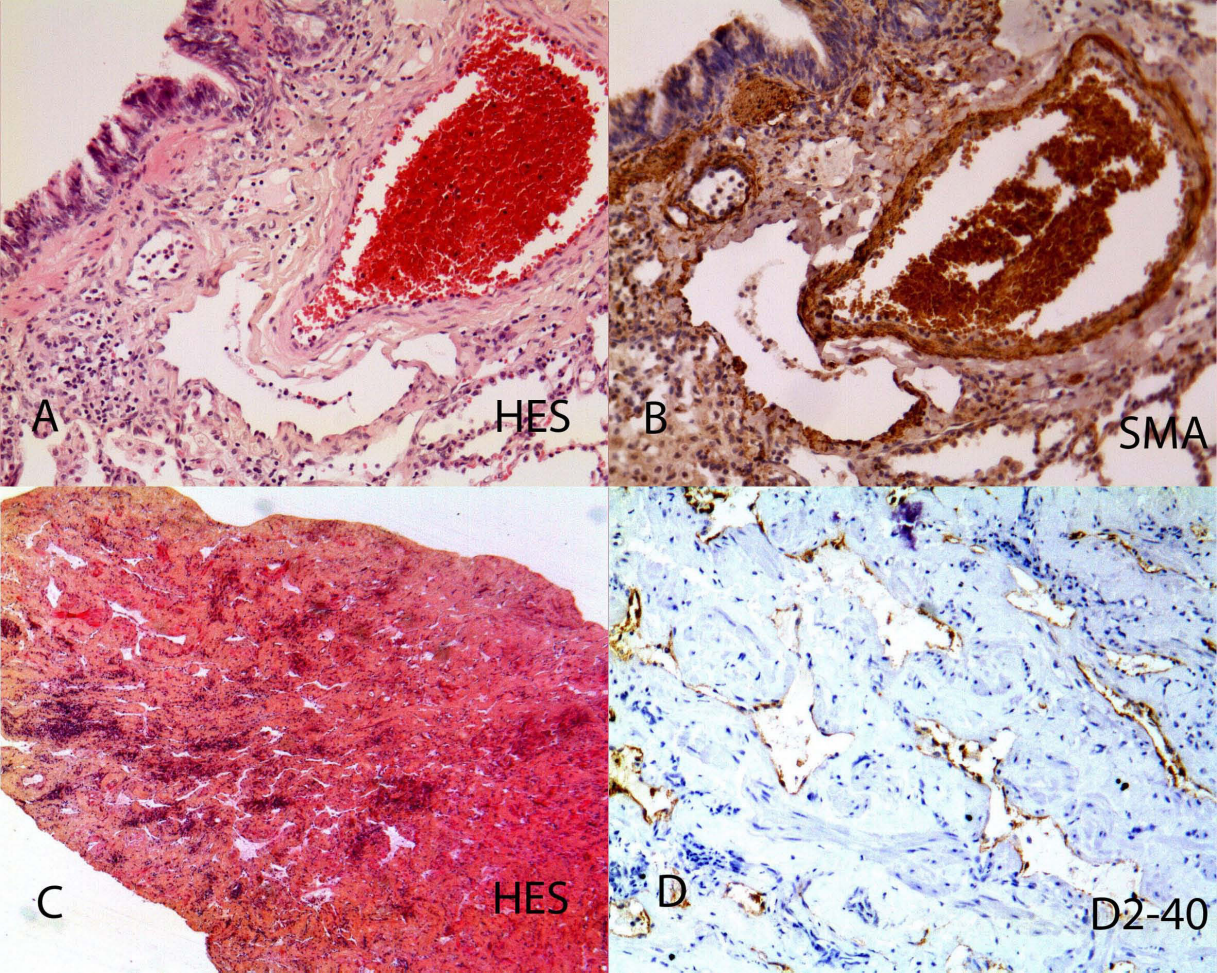
**Pathological findings: Lung locations**. A: Peribronchiolar dilated lymphatics (HES × 400). B: Foci of smooth muscle actin positivity alongside dilated lymphatic (SMA × 200). C: Parietal biopsy at low magnification showing its thickening, with numerous lymphatic cavities and inflammation (HES × 100). D: Higher magnification of the D2-40 lining of vascular cavities with some smooth muscle bundles in between (SMA × 200).

Pathological specimens were reviewed in 7 cases (figure 5 and 6). Bone specimen (humerus, femur, scapula and skull) were recovered. The humerus and scapula specimens showed endothelialized sinusoids replacing the bone, with no osteogenesis and disappearance of the bone marrow. A small femur biopsy showed a focus of osteoclastic hyperactivity and the skull specimen included the wall of an endotheliform cavity. Lung and pleural specimens were available in two cases and showed numerous abnormal lymphatic cavities with accompanying smooth muscle bundles. Skin biopsy was done in one case and showed an hypodermic focus of aggregated lymphatic cavities with some smooth muscle differentiation. A spleen specimen was available in one case and showed a network of endothelialized sinusoids lined by some smooth muscle bundles. Immunohistochemistry was done and showed D2-40 positivity of the endothelial lining, and a continuous CD31 positive lining of the spleen cavities.

### Therapeutic management

In addition to symptomatic treatments, three therapeutic approaches were attempted, namely radiotherapy, cytostatic drugs (including vinca alkaloids and steroids), and antiangiogenic drugs (α interferon, bisphosphonates, thalidomide and bevacizumab) [Table S2, Additional file 1]. Treatment was tailored to the individual patients. We analyzed treatment efficacy according to the affected organs, as each site of involvement presents different targets and therefore different methods of assessment.

Management of patients with symptomatic pulmonary involvement (n = 9) consisted of routine pleural drainage, with octreotide infusion in 4 cases and surgical shunting in 3 cases. In one case (case #13) drainage alone was sufficient to prevent further relapses. Various curative treatments were attempted in refractory forms: radiotherapy of the chest wall or a lung half-field (n = 4), chemotherapy with vinca alkaloids (vincristine and vinblastin) (n = 3), and interferon alfa, with or without a bisphosphonate or thalidomide (n = 4) and finally bevacizumab in one case. Pleural radiotherapy was administered at individually tailored doses. Four cases of pleural effusion were treated with chest irradiation at doses of 49.5 Gy in case #12 (unilateral), 18 Gy (right)+ 12 Gy (left) in case #11, 18 Gy in case #8 (unilateral) and 8.5 Gy in case #14 (unilateral). The three patients treated with the highest doses have had no recurrent effusion during more than 8 years of follow-up, but they have severe orthopedic sequelae. Patient #8, after a marked clinical improvement at 6 months, remained stable until the recurrence of pleural effusion with intrapulmonary lymphangiectasis 8 years later, but the pleural effusion relapsed at this time. This relapse was refractory to a combination of α interferon and thalidomide. The patient treated with a lower dose (8.5 Gy) died within two months, with no improvement in the pleural effusion. Two patients were treated with vinca alkaloids (vincristine (n = 2) and vinblastine (n = 1); at least six injections), combined with oral steroids (40 mg/m^2^/d for 4 weeks). No improvement was observed in either case. Four patients received α interferon, one in combination with zoledronate, one in combination with pamidronate and one in combination with thalidomide. The patient treated with α interferon alone (case #15) had systemic toxicity, leading to treatment withdrawal after 83 days with no improvement. The patient treated with α interferon and pamidronate (case #19) showed no improvement. The patient treated with α interferon and thalidomide (case #8) was refractory and died at month 2. Finally, only the patient (case #18) treated with α interferon and zoledronate showed an improvement in pleural effusion, at month 3, but the pleural effusion relapsed at month 9 and was not sensitive to bevacizumab.

Management of seven patients with skin and subcutaneous lesions or subcutaneous tumor was also very diverse. Three patients received radiotherapy. In patient #11, a subcutaneous lesion of the left hemithorax disappeared after 6 months of radiotherapy (12 Gy) prescribed for pleural effusion, while patient #14 and #15 showed no improvement following 8.5 Gy and 15 Gy. One patient (#9) received the standard treatment for Langerhans histiocytosis (12 injections of vinblastine and steroids 40 mg/m^2^) for 6 months, to no effect. Two patients were treated with α interferon alone: Patient #15 was treated for 83 days with no measurable effect, and patient #16 was treated for at least 3 months and had no measurable improvement. Two patients received a bisphosphonate, with or without α interferon: Patient #4 (treated with zoledronate 0.05 mg/kg IV every 3 months) had no measurable improvement. Patient #9 was treated with etidronate (400 mg/d) and α interferon (2.5 × 10^6 ^U/m^2 ^three times a week), and the supraclavicular and cervical extension appeared to remain stable during 9 months of follow-up.

Involved bone was a target of systemic treatments in 10 patients but the short-term response was difficult to assess because it was usually asymptomatic, except in case of fracture. In addition to local treatments, only α interferon and/or bisphosphonates were tried in these ten cases. In seven cases (#2, #3, #4, #9, #18, #19, #20) no bone complications have occurred, but these patients have less than two years of follow-up. In patient #1, treated with α interferon (1.5 × 10^6 ^U five times a week then tapered to three times a week) secondarily combined with pamidronate, no bone complications have occurred with more than two years of follow-up. Finally, new fractures occurred during long-term pamidronate therapy in patient #6 and during long-term pamidronate/zoledronate and α interferon in patient #7.

Embolization (case #13) and sclerotherapy (case #16 and #17) were also attempted in three cases but only patient #17 showed an improvement. Surgery aimed at resecting all angiomatous tissue was never attempted in this series.

### Complications, outcome and prognosis

The disease progressed either gradually or discontinuously, with periods of stabilization and symptom disappearance followed by new complications. However, it was difficult to identify disease progression formally, as regular evaluations were not performed.

Nine patients had respiratory complications leading to respiratory distress, which was fatal in five cases. Respiratory distress was secondary to pleural effusion or to an exacerbation of pulmonary lymphangiectasis. Spinal complications, including vertebral collapse, occurred in 10 patients. They were generally mild but were accompanied by spinal cord damage in two cases, with one death by cervical cord section and one case of permanent paraplegia. Pathological fractures occurred in 8 patients. Finally, one patient had recurrent meningitis due to an osteomeningeal breach secondary to skull base involvement. Biological signs of disseminated intravascular coagulation were observed in five cases and lasted several months.

Six patients died (29%). The survival rate 10 years after onset was 71% (95%CI 43-87%) but the curve has not yet plateaued. The six deaths occurred between 3 months and 16.9 years after onset (median 2.4 years). The causes of death were pulmonary involvement (5 cases) and spinal involvement (1 case).

Four patients are in persistent clinical remission, with an active phase of 1 year (#11), 4 years (#12), 10 years (#7) and 13 years (#13). Three of the four patients in remission have severe sequelae, consisting of radiotherapy-induced scoliosis in two cases and paraplegia secondary to vertebral collapse in one case.

## Discussion

This retrospective survey identified 21 cases of aggressive vascular abnormalities of bone in children over a 21-year period in France. Despite the small number of cases, this is the largest pediatric series published to date. We used a national network of specialists in pediatric hematology-oncology and including pediatric orthopedists, as most patients present with what resembles a bone tumor and are thus referred to such specialists. Although we cannot be sure we identified all French cases, it is unlikely that the true number of cases was much higher, and the prevalence of the disease probably corresponds to that of extremely rare conditions, i.e. between 0.1 and 1 case per million children less than 15 years of age. There was a male predominance in our series, as reported in the literature [2,14]. We found no familial cases and no consanguinity. No intercurrent disorders were present in 20 of the 21 patients. The association with dominant familial exostosis is noteworthy, even if no clear link has been found between the two disorders. The diagnosis of the disease in 3 cases after bacterial infections of the same serous membrane is intriguing, but no conclusion can be drawn from this observation alone. Most patients presented with symptoms linked to bone lesions (fractures) or to pulmonary involvement. In three cases the initial diagnosis was Langerhans histiocytosis and the patients received the relevant treatment before the diagnosis was corrected (notably in view of the lack of therapeutic efficacy).

This series comprised multifocal forms with cystic intraosseous lesions seen on imaging studies (n = 16), and localized forms (n = 5), corresponding to the two previously identified forms of « osseous angiomatosis », namely multifocal cystic osseous angiomatosis for multifocal forms and aggressive massive osteolysis (aka Gorham-Stout disease) for unifocal forms. Careful appraisal of the patients' medical history and imagery shows, however, that there is no clear basis for the distinction between these two forms. The 5 patients initially considered as having localized disease resembled the patients with multifocal forms, with respect to demographic characteristics and outcome (especially secondary pulmonary extension and mortality). One of our patients (case #14) was considered to have localized involvement (thoracic cage), complicated by pleural effusion, but autopsy revealed the presence of abnormal vascular tissues in the skull and pelvis and urinary bladder. Moreover, not all the patients had radiographic studies of the entire skeleton, as in some "localized" forms reported in the literature. Thus, in the absence of precise biological, pathological or even radiological criteria, we suggest that all patients should be considered to belong to a single nosologic entity. In future studies of aggressive vascular malformations of bone, the diagnostic work-up should systemically include all bones and lung CT.

Diffuse bone demineralization was observed in ten of the eleven assessable cases. This phenomenon has also been reported in the literature [19] and supports the current view that the osteolysis is related to osteoclast hyperstimulation [15,20,21].

Pathological specimens were reviewed in 7 cases. The bone lesions were composed of abnormal non fenestrated sinusoids with continuous wall CD31 positivity. Lung lesions showed subpleural and peribronchial dilated lymphatic cavities, sometimes with smooth muscle bundles in the walls. Spleen lesions showed disseminated cystic cavities of non fenestrated CD31-positive sinusoids with surrounding smooth muscle. Skin lesions were mainly hypodermic, with foci of cystic lymphatic D2-40 positive and venula CD31 positive abnormal aggregation. In summary, aggressive vascular abnormalities of bone lesions mainly consisted of capillarized sinusoids in bone marrow and spleen and abnormal lymphatics with smooth muscle lining in lung, pleura and skin. A recent study showed that these capillarized sinusoids are CD 105 (endoglin)-positive, like the bone of growth plate capillaries [22]. Among aggressive vascular abnormalities of bone, it is difficult to distinguish between venous and lymphatic subtypes, as suggested in Bruder's recent review article [3]. Likewise, the literature contains numerous borderline forms between predominantly venous and predominantly lymphatic forms. Immunohistochemical studies, especially those focusing on CD31 (an endothelial marker), CD34 (expressed by blood vascular endothelial cells), D2-40 and LYVE-1 (lymphatic endothalial cells), markers of vascular proliferation such as the MIB1 proliferative index and VEGF expression within the lesions, would appear to be more useful for subdividing, or on the contrary uniting, these pathological entities. We recommend grouping together these disorders under the term « aggressive vascular abnormalities of bone », instead of the older term « angiomatosis of bone», as the « oma » suffix is suggestive of tumor proliferation.

In this study, neither chemotherapy with spindle poisons nor steroid therapy at a dose of 40 mg/m^2 ^was beneficial. Thirteen patients received α interferon (n = 8) and/or bisphosphonates (n = 10). Only six cases treated with α interferon, associated with bisphosphonates in 3 cases, have been reported in the literature [Table S3, Additional file 1] [9,10,23-26]. We observed only short-term efficacy in one patient with pleural effusion (#18), whereas the literature describes four cases of successful treatment and one failure [Table S3, Additional file 1]. The value of α interferon-bisphosphonates combinations in this setting remains to be determined. These treatments, especially after prolonged administration, appeared more effective for preventing bone complication and limiting the osteolytic process, as observed in two cases (#1 and #7), but the other cases have insufficient follow-up. Currently, there is no clinical basis on which to choose a particular bisphosphonate.

High-dose thoracic radiotherapy (at least 18 Gy) had a durable impact on pleural effusions, but two children thus treated developed severely incapacitating scoliosis and a relapse was observed in 1 of 3 cases after 8 years. In contrast, a dose of 8.5 Gy was not effective. Thus, it seems that high doses must be used in this setting. Indeed, other authors have recommended doses between 25 and 45 Gy [27-30]. Fractionated bone radiotherapy at a total dose of 25 to 40 Gy can stop the osteolytic process [31-34], but failures have also been published [2,23,35,36]. None of our patients underwent surgery aimed at resecting all the vascular abnormal tissue. Surgical treatments had variable results, and bone grafts during the active phase of the disease have a tendency to be resorbed [11,31,37].

In our series the vital prognosis depended on pulmonary and spinal involvement. Special attention must be paid to localized forms affecting the thoracic cage, as they carry a high risk of pulmonary complications. Pulmonary complications such as pleural effusion have already been reported in the literature, with an estimated incidence of between 17% and 25% [9,38]. In contrast, 47% of our patients presented with radiographic and/or clinical signs of pulmonary lymphangiectasis or pleural effusion. We observed five cases of pulmonary lymphangiectasis, characterized in four cases by respiratory failure and associated with pleural effusion; no clinical manifestations occurred in the last case.

## Conclusion

Aggressive vascular abnormalities of bone are rare but potentially life-threatening disorders of unknown etiology. Localized forms and multifocal cystic bone forms are very similar, and there seems to be no reason to consider them as distinct entities. Pulmonary involvement is a major prognostic factor. The efficacy of antiangiogenic treatments and of third-generation bisphosphonates such as zoledronate is unclear and must be further evaluated. Molecular biology-based approaches, including immunolabelling of bone lesion samples, should help to understand these disorders and to orient the choice of treatment.

## Competing interests

The authors declare that they have no competing interests.

## Authors' contributions

The study was designed by SH and JD. Data management was coordinated in France by SH. MLM, FJ, MB, MGY, BC, MZ, YB, CC, PC, CBB, JCL, MQ, LBG, PM, JB, IP, DW, YR, VH, VF, HDL, JL, JW, RE, BF, and JD were responsible for patient management. The manuscript was written and approved by all the coauthors.

## Supplementary Material

Additional file 1 **Table S1, Table S2, Table S3**. Table S1 - Disease presentation - mean features of 21 patients with aggressive vascular abnormalities of bone. Table S2 - Therapeutic management and outcome of 21 patients with aggressive vascular abnormalities of bone. Table S3 - Summary of the literature on α interferon and/or bisphosphonate therapy in aggressive vascular abnormalities of bone Click here for file
