# Supplementary material for: Retrospective French nationwide survey of childhood aggressive vascular anomalies of bone, 1988-2009
Source: Orphanet J Rare Dis. 2010 Feb 3;5:3. doi: 10.1186/1750-1172-5-3 (PMC2830950; doi:10.1186/1750-1172-5-3)
Supplement: Additional file 1 — Table S1, Table S2, Table S3. Table S1 - Disease presentation - mean features of 21 patients with aggressive vascular abnormalities of bone. Table S2 - Therapeutic management and outcome of 21 patients with aggressive vascular abnormalities of bone. Table S3 - Summary of the literature on α interferon and/or bisphosphonate therapy in aggressive vascular abnormalities of bone [file 1750-1172-5-3-S1.DOC]

**Table S1:**

Legend: F: Female M: Male; MRI: Magnetic resonance imaging; MD: Missing data; ND: Not done; DIVC: Disseminated intravascular coagulation; Lymphang: Pulmonary lymphangiectasies; * radiography of entire skeleton

| UPN | Age at first symptom / age at diagnosis  (years) | First symptoms | Gender | Overall number of distinct bone involved | Lung involvement | Extra bone and extra lung involvement | Fractures, if yes number of episodes | Vertebral collapse | Aspect of bone lesions on MRI | Osteopenia |
| --- | --- | --- | --- | --- | --- | --- | --- | --- | --- | --- |
| 1 | 9.6 / 9.8 | X rays for a facial trauma secondary to a malaise | F | 11* | No | Spleen | No | No | Numerous bone lesions, well limited hypo T1, hyper T2, with weak gadolinium enhancement | Yes |
| 2 | 16.4 / 16.9 | Chronic pain of iliac bones | M | 3* | No | No | No | No | Hyper T2 lesions, peripheral gadolinium enhanced | Yes |
| 3 | 2.9 / 3.5 | Recurrent bacterial meningitis - meningeal breach | M | 11* | No | Spleen, latero cervical soft tissues | No | No | Bone lacunes with liquid signal and peripheral gadolinium enhancement. Bone lacunes are located inside fatty abnormal signal | ND |
| 4 | MD / 4.3 | Bone fracture | M | 10* | No | Spleen, subcutaneaous, left hemi thorax soft tissues | 1 | No | Centro-medullar and cortical bone cystic lesions with liquid signal. Ring shaped peripheral gadolinium enhancement with secondary filling | No |
| 5 | MD / 1.4 | Persistent limp | M | 4* | No | Subcutaneaous, right limb soft tissues | 1 | No | Bone lesions hypo T1, hyper T2 and hyperSTIR | ND |
| 6 | 3.45 / 3.5 | Bone fracture | M | 13* | No | No | 4 | Yes | MD | Yes |
| 7 | 8.4/ 8.7 | Bone fracture | M | 11* | No | Spleen | 3 | Yes | Bone cystic lesions | Yes |
| 8 | 5.6/ 6.3 | Dyspnea | F | 3 | Lymphang Pleural effusion | DIVC | No | Yes | MD | Yes |
| 9 | 4.7 /15 | Bone fracture | F | 6 | Lymphang | Sus clavicular and cervical soft tissues | 5 | Yes | MD | ND |
| 10 | MD / 11.9 | Dyspnea | M | 3* | Lymphang  Pleural effusion | Mediastin,  DIVC | No | Yes | Vertebral Heterogeneity hypo T2 or hyper T2 | Yes |
| 11 | 3.8 / 3.9 | Dyspnea | M | 5* | Pleural effusion first hemorragic then chyleous | Spleen, subcutaneaous, left hemi thorax soft tissues,  DIVC | No | Yes | MD | Yes |
| 12 | 12.7 / 12.8 | Dyspnea | F | 3* | Pleural effusion (hemorrhagic) | Latero vertebral soft tissues | No | No | T1 hyper signal dorsal vertebra | ND |
| 13 | 11.5 / 12.9 | Chronic lombalgia | M | 2 | Chylothorax | No | No | Yes - Paraplegia | Left para vertebral mass hypo T1 hyper T2 | ND |
| 14 | 11.2 / 11.3 | Subcutaneous and skin dorso lombar tumefaction | F | 5 | Chylothorax | Skin and subcutaneaous in the dorso lombar region, bladder | No | No | MD | ND |
| 15 | 1.38 / 1.4 | Dyspnea | M | 12* | Lymphang  Chylothorax | Spleen, mediastin, peritenous, subcutaneaous, soft tissue left flank | 1 | Yes | Vertebral hyper signal T1 and T2 | ND |
| 16 | 9.96 / 10.4 | Latero cervical tumefaction | M | 4 | No | Latero cervical soft tissues | No | Yes -  Death | Occipito- cervical hyper T2 mass  suggestive of lymphangiectasies | ND |
| 17 | Birth / 7 | Latero cervical tumefaction hemangioma | F | 4 | No | Spleen,  perinatal cervical hemangioma, subcutaneaous | No | No | CT scan only | ND |
| 18 | 13.2 / 13.6 | Dyspnea | F | 8 | Lymphang Chylothorax | DIVC | No | No | CT scan only | Yes |
| 19 | 0.1 /0.8 | Dyspnea | M | 3 | Lymphang Chylothorax | DIVC | No | No | MD | Yes |
| 20 | 9 /11 | Vertebral collapse cyphosis | F | 8 | No | No | 1 | Yes | T2 hyper signal dorsal vertebra | Yes |
| 21 | 0.6 /1.8 | Inguinal tumefaction | M | 8* | No | Spleen, subcutaneaous, soft tissues | 1 | No | Bone lesion hypo T1, hyper T2 and hyper STIR | ND |

**Table S2:**

| UPN | Therapy before the diagnosis and response | Target organ  for therapy | First line therapy | Time from therapy outset and last evaluation or therapeutic change  = Initial response | | Secondary Line therapy | Delays from therapy change and evaluation  = response to second line | sequelae | Outcome  Follow-up since diagnosis /  Age at last Follow-up (years)  Vital status |
| --- | --- | --- | --- | --- | --- | --- | --- | --- | --- |
| 1 | Steroid / vinblastine  1 pulse | Bone | Year 1: α Interferon 1,5 M U *5/w then tapered down 3*/week | 1 year  No fracture but no bone reconstruction | | Year 2: α interferon associated with Pamidronate | 1 year  No fracture, no bone reconstruction but better bone density |  | 2.2 years / 12 years  Alive |
| 2 |  | Bone | Pamidronate  1mg/kg 3 days/3 months | 1.1 years  No fracture | |  |  |  | 2.5 years / 19.4 years  Alive |
| 3 |  | Bone | Pamidronate  1mg/kg 3 days/3 months | 0.5 years  No modification of bone aspect  No fracture | |  |  |  | 2.1 years / 5.6 years  Alive |
| 4 |  | Sub cutaneous tumor  Bone | Zoledronate  0,05 mg/kg/3 months | 0.9 years  No effect on SC tumor  No fracture on therapy | |  |  |  | 1.9 years / 6.2 years  Alive |
| 5 |  | Bone | No specific therapy | Progression of bone lesions volume by year 4.5 | |  |  |  | 4.9 years / 6.3 years  Alive |
| 6 |  | Bone | Pamidronate 0,5mg/kg/d x 3 days every 3 months | 4.5 years  2 fractures | |  |  |  | 6.6 years / 10.1 years  Alive |
| 7 |  | Bone | Pamidronate 0,7mg/kg/d x 3 days every 3 months | 5 year1/2  One fracture | | α Interferon  + Zoledronate 0.05 mg/Kg/3 months | 2.2 years  1 fracture |  | 10.7 years / 19.4 years  Alive |
| 8 | Prednisone Vinblastine  Purinethol then Vincristine (No efficacy by month 9) | Pleural effusion  Lymphangiectasies | Lung half field radiotherapy  18 Gy | Improvement by the end of year 1 | | Relapse year 8  Treated by α interferon and thalidomide | No efficacy – death by month 2 | Chronic respiratory insufficiency after radiotherapy | 10.6 years / 16.9 years  Deceased |
| 9 | Vinblastine, steroid  (No efficacy by month 12) | Subcutaneous tumor  Bone | α Interferon 2,5 M U/m^2^ *5/w  + Etidronate oral | At month 3 =  stability of SC lesion  No fracture | |  |  |  | 3 years / 18 years  Alive |
| 10 |  | Pleural effusion | Vincristine | No efficacy | |  |  |  | 2.3 years / 14.2 years  Deceased Respiratory distress |
| 11 |  | Pleural effusion  Sub cutaneous tumor | Radiotherapy18+12 Gy | Pleural effusion and SC tumor controlled by month 3 |  | |  | Severe scoliosis | 13.3 years / 17.2 years  Alive |
| 12 |  | Pleural effusion | Radiotherapy 49.5 Gy | Pleural effusion controlled by month 3 |  | |  | Severe scoliosis  and psychosis | 15 years / 27.8 years  Alive |
| 13 |  | Pleural effusion  Bone | Symptomatic therapy for pleural effusion  + Vertebral Embolisation | Pleural effusion controlled by month 3  Serious vertebral collapse |  | |  | Paraplegia/ chronic back pain | 16,4 years / 29.3 years  Alive |
| 14 |  | Pleural effusion  Skin, sub cutaneous tumor | Radiotherapy 8.5 Gy | No efficacy on pleural effusion skin and SC tumor |  | |  |  | 0,3 years / 11.6 years  Deceased bilateral chylothorax |
| 15 |  | Pleural effusion  Subcutaneous tumor | α Interferon for 83 days 1,5 MU x1 until 9 MU/days | No efficacy on pleural effusion and SC tumor | Radiotherapy 15 grays (localized on sub cutaneous tumor) | | No efficacy |  | 16.9 years / 18.3 years  Deceased bilateral chylothorax |
| 16 |  | Subcutaneous tumor | Per cutaneous sclerotherapy | No efficacy on SC tumor | α Interferon (at least 3 months) | | No efficacy |  | 3 years / 13.4 years  Deceased (cervical medullar compression) |
| 17 |  | Subcutaneous tumor | Sclerotherapy of sub cutaneous tumefaction | No relapse of SC tumor by year 8 |  | |  |  | 11.3 years / 11.4 years  Alive |
| 18 |  | Pleural effusion  Bone | α Interferon  + Zoledronate 0.05 mg/kg/3 months | Pleural effusion controlled by month 3 but relapse by month 9  No fracture | Bevacizumab | | No efficacy but given at end stage |  | 0.9 years / 14.5 years  Deceased bilateral chylothorax and H1N1 viral infection |
| 19 |  | Pleural effusion  Bone | α Interferon  + Pamidronate  1mg/kg 3 days/3 months | No efficacy on pleural effusion  No fracture |  | |  |  | 0.7 years / 1.5 years  Alive |
| 20 |  | Bone | Pamidronate  1mg/kg 3 days/3 months | 0.5 years  No fracture on therapy |  | |  | Cyphosis | 0.6 years / 11.6 years  Alive |
| 21 |  | Subcutaneous tumor  Bone | No specific therapy |  |  | |  |  | 0.3 years / 2.1 years  Alive |

Table S3:

| Author  Reference | Age / Sex | Disease extension and target for therapy | Interferon / Dose | Bisphosphonates | Associated drugs | Schedule | Indication | Response | Duration of therapy | Outcome after therapy | Adverse events |
| --- | --- | --- | --- | --- | --- | --- | --- | --- | --- | --- | --- |
| Takahashi [10] | 2 / F | Multifocal bone and chylothorax | α interferon  1,5 M U | No | Prednisone 5mg/j | Daily for 2 months then tapered down to 1,5 M U /w | Chylothorax | Chylothorax and hemangioma cure | 14 months | 10 months | Fever |
| Hagberg [23] | 19 / M | Localized bone and chylothorax | α 2b interferon  3 M U then 5 M U | Clonodronate  oral | No | Daily | Chylothorax | Cure by week 6 without relapse | 19 months (until last update) | 19 months (until last update) | Auto immune hypothyroïdy |
| Pfleger [9] | 18 / M | Multifocal bone and chylothorax | α 2b interferon  3 M U | Clonodronate  oral | No | Daily | Chylothorax | No assessable | 10 days / withdrawn related to severe thrombocytopenia |  | Thrombocytopenia |
| Pfleger [9] | 18 / M | Multifocal  bone and chylothorax | PEG α interferon 2b  50 μg | No | No | Once /week | Chylothorax | Cure without relapse | ND | 12 months | No |
| Somoza Argibay [24] | 5 / M | Multifocal bone and chylothorax | α 2b interferon  2,2 M U | No | No | Daily | Chylothorax | Improvement | ND |  | No |
| Hagendoor [25] | 17 / M | Localized bone | α 2b interferon  3 M U  then 6 M U | Pamidronate  intra venous | Thalidomide | Daily | Severe Osteolysis | Progression / failure | ND |  | No |
| Kose [26] | 9 / F | Multifocal  bone and chylothorax | α 2b interferon | No | No | 3/week | Chylothorax | Improvement | 6 months | Progression of bone lesion but stability of lung involvement | No |
